# Disaster Risk Resilience in Colima-Villa de Alvarez, Mexico: Application of the Resilience Index to Flash Flooding Events

**DOI:** 10.3390/ijerph16122128

**Published:** 2019-06-16

**Authors:** Mendoza-Cano Oliver, López-de la Cruz Jesús, Pattison Ian, Martinez-Preciado MA, Uribe-Ramos Juan Manuel, Edwards R. M., Ramírez-Lomelí Cesar Ivan, Rincón-Avalos Pedro, Velazco-Cruz Jorge A

**Affiliations:** 1 Faculty of Civil Engineering, University of Colima, Colima 28400, Mexico; jlopez71@ucol.mx (L.-d.l.C.J.); I.Pattison@lboro.ac.uk (P.I.); martinez_miguel@ucol.mx (M.-P.M.); jmuriber@ucol.mx (U.-R.J.M.); cramirez6@ucol.mx (R.-L.C.I.); princon0@ucol.mx (R.-A.P.); jvelazco@ucol.mx (V.-C.J.A.); 2 School of Architecture, Civil and Building engineering, Loughborough University, Loughborough LE11 2UT, UK; r.m.edwards@lboro.ac.uk; 3 5G Research Centre, Woflson School, Loughborough University, Loughborough LE11 3TU, UK

**Keywords:** flooding, risk perception, resilience, Likert

## Abstract

Resilience is an indicator of the ability of systems to withstand disruption within acceptable degradation parameters and also their recovery time. It is essential for public policies to understand how the population reacts to a particular risk. In this paper we have performed a study that quantitatively measures perceptions of flooding and resilience to flooding in the city of Colima-Villa de Alvarez, Mexico 2018–2019. A resilience index has been applied to ten zones of the city. In our research we assessed risk perception through a city-wide survey with questions based on a Likert scale. An analysis was performed on public knowledge of the existing security protocols for floods and evaluated the public perception of the availability of critical services, such as fresh water, electricity, food, drainage, communications and public transport during a flash flood events. This research has identified populated low resilience zones that can be considered as priorities for resource and effort to mitigate floods and their impacts. The novel resilience index developed in this work can also be applied to other type of risk that humans face and used as a basis for discussions about urban resilience.

## 1. Introduction

Hydrometeorological events such as floods, hurricanes, and coastal storm surges can be a direct threat to human lives and impact livelihoods and cause damage to crops, businesses, and critical infrastructure. The ensuing floods have a fast-hydrological response, recognized by hydrographs showing steep rising and falling limbs and, accordingly, short lag times. This results in what are known as “flash floods” due to their rapid onset, that is, with only a few hours between rain falling and flooding [1,2,3,4]. The impacts of these type of flood events are often underestimated in terms of their cascading effects on critical infrastructure. and poor upstream management decisions have exacerbated the risks on communities. 

Public perception of resilience and participation in resilience policy are both important components at all stages of the implementation of water related policies, particularly related to flood management. A combination of structural flood defense infrastructure and non-structural measures, such as Natural Flood Management [5] and flood forecasting and warning, have become essential elements in flood control policies, demanding more collaboration on the part of the affected population than traditional structural measures [6]. 

Resilience to floods is a useful concept to study in terms of the capacity of households to cope with, and adapt to floods. Knowledge of the resilience of households to floods can help disaster risk managers to design policies for living with floods [7]. The Flood Resilience Index was built to support the decision-making process in choosing design alternatives that improve flood control responses in future scenarios that surpass design standards [8,9]. 

Risk perception, and individual and collective attitudes in managing and adapting to hazards are strongly influenced by social and cultural factors that reflect the values and history of a community [10,11,12]. The relationship between what people know and perceive about risks and how risk perception can play in inhibiting or encouraging adaptive actions by individuals and institutions alike has been the subject of multiple research over various disciplines [13,14,15,16].

The aim of this research is to understand how the perception and response to natural hazards such as floods in an urban area can be translated into a resilience index for preparedness for flash flooding events. In this article, we present the perception of risk for flash flooding in the area of Colima-Villa de Alvarez in Mexico, and also the resilience index for the areas in which a flash flooding event has occurred. This resilience index has allowed the quantification and identification of the environmental impacts of this type of event. The index is also a baseline for further studies. We expect that this type of knowledge will contribute not only to new research about flooding in Mexico but also to a policy agenda that will be able to respond to future challenges in global flash flooding events.

## 2. Materials and Methods

### 2.1. Case Study

**Figure 1:**
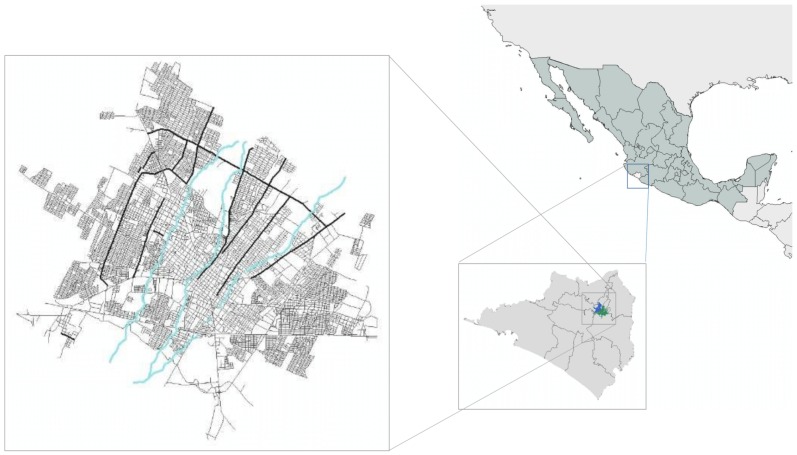
Colima-Villa de Álvarez urban zone.

Flooding in Latin America and the Caribbean is widespread and is probably among the most commonly experienced disaster types [17]. Colima-Villa de Alvarez metropolitan area (Figure 1) was chosen as the case study due to the presence of flash flooding events in recent years, and as it is a useful reference for most cities in Latin America. 

**Figure 2:**
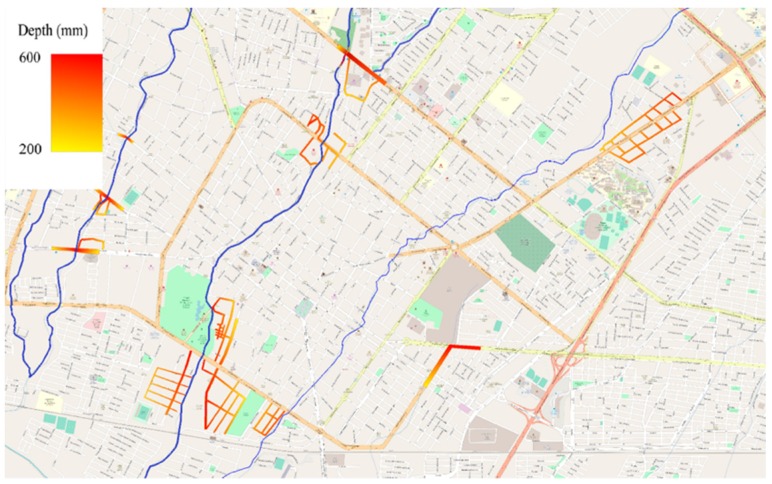
Flooded zones (streets) in Colima-Villa de Alvarez area by flash flooding events.

The Colima-Villa de Álvarez metropolitan area has a population of 334,240, with an Annual Growth Rate of 2.1% [18]. There are several rivers flowing through the city, the Colima, Manrique and Pereira [19]. In the last few years events (2000–2018) of flash flooding have occurred in different zones, one of them occurred on September 2018 (Figure 2) when an intense rain that lasted 20 minutes had several effects on many points of the cities. In some cases, the water reached 800 mm of depth with the respect of the ground level.

### 2.2. Objective and Questionnaire

Psychosocial investigations took place in the city of Colima-Villa de Alvarez, with a focus in zones where previous problems of flash flooding have occurred. Those zones are shown in Figure 2, and the focus of the research was how resilient the population is, as well as the perception of the people and the response time of the emergency support agencies, and also the rehabilitation of basic services. This survey consisted of a ‘‘face-to-face’’ application of a standardized questionnaire with a total number of 13 questions. 

The construction of the items of this questionnaire was made with help from a group of experts, through qualitative methods (focus-group). To check validity, the initial survey was applied to a pilot sample of 50 persons, some of the questions having suffered slight modifications afterward. Taking into consideration the complexity of the topic and the need to obtain a level of information as large and as diverse as possible, three themes were identified to gather information on: the awareness of the protocols of the National and State Civil Protection Agency before extreme hydrometeorological events linked to flooding, the behavior and reaction during a flood inundation event; and the evaluation of risk towards material goods or people. The items have been coded with answers on a Likert scale of 2, and 6 levels (0 being the lowest and 5 being the highest). The questions were based on a literature review of risk perception in a remediation context, discussions with a technical expert team consisting of an environmental economist, and a social scientist, as well as a pilot survey. 

A total of 273 respondents were included in the analysis. A random sampling strategy was used within the flooded zones. The subjects’ attitude has been, in most cases, friendly, some of them showing interest in the issue presented. The study was made via an in-person survey conducted two to four days per week between December 2018–January 2019. For a low percentage of subjects interviewed, we encountered high susceptibility or even hostility as they refused to be interviewed. This aspect could be interpreted from the perspective of the violence occurring in the zone, and high vulnerability of those populations. 

### 2.3. Risk Perception and Resilience Analysis

People are exposed to hazards, so the community and broader society are directly impacted. However, the perceived risk can be either heightened or diminished by exposure and societal influences. Risk attenuation occurs when experts indicate that an area and community are at risk, whilst the community does not and pays comparatively little attention to that risk. Risk perception is also linked to flood memory and if they have been flooded previously. In this study, the risk analysis was conducted to assess how individuals perceived their risk of a flash flood event occurring again; therefore, putting the perceptions of flooding into perspective. Results of the risk analysis were also used to calculate the flood resilience index for the area.

We had 10 zones in the study area. Respondents were asked to rate whether critical services were available during floods on a scale from 0 which represented never available (i.e., “no services available, so high risk assumed”); 3 was a moderate option (i.e., “moderately risky”); and 5 represented availability of services (i.e., “no risk”). Similar scales have been successfully used to assess perceived environmental and health risks [11,20,21,22]. We also asked the respondents about actions to take during a flood, and how they gained information about a flood event. A percentage distribution Likert-Type survey data analysis [23] was conducted for this study to assess perceived likelihoods of risks to listed flash flood events. 

We then calculated the resilience index using the platform Anaconda Navigator, which is a repository of different programming languages included in R as RStudio [24]; taking into account the characteristics of urban functions that are defined in the perception questionnaire. The requirements are related to the provision of different building (water, energy, transportation, communication) and to investigate the operational function during and after a flood. Then from the perception survey, the availability levels of urban functions were set as follows: 

Availability level 0: Not available, 

Availability level 1: Poor available; major interruptions; 

Availability level 2: Low availability; interruptions provide minimum availability; 

Availability level 3: Medium; small interruptions that are tolerable for small flood durations; 

Availability level 4: Medium high; interruptions that are tolerable for long flood durations: 

Availability level 5: Requirements fully provided. 

Finally, we calculated the Flood Resilience Index (FRI) = Availability level/number of functions of characteristics.

The specific questions we used to calculate the resilience index were: “How much are drinking water services available before a flood occurs and, after it has occurred?”, “Are communications services available before a flood occurs and after it?”, “Is the transportation service available before a flood occurs and, if necessary, after it has occurred?”, “How are drainage services available before a flood occurs and, if so, after it occurs?”, How much are food services available before a flood occurs and after?”, “To what extent are urban services adequate before a flood occurs and, if necessary, after it has occurred?” and “To what extent are urban services adequate before a flood occurs and, if necessary, after it has occurred?”. 

### 2.4. Limitations of the Proposed Index

We developed the flood resilience index with the ability to assess all indicators objectively. The outcome indicators were developed from actions in the flood risk management cycle and depend on some assumptions. The proposed measurement of indicators relies on weights (assigned for each variable). Some limitations related to providing a quality measure of the process are possible since weights are used to intensify the scores in the assessment.

## 3. Results

### 3.1. Public Knowledge of Security Protocols of the Civil Protection Agency Related on Floods and Knowledge

**Figure 3:**
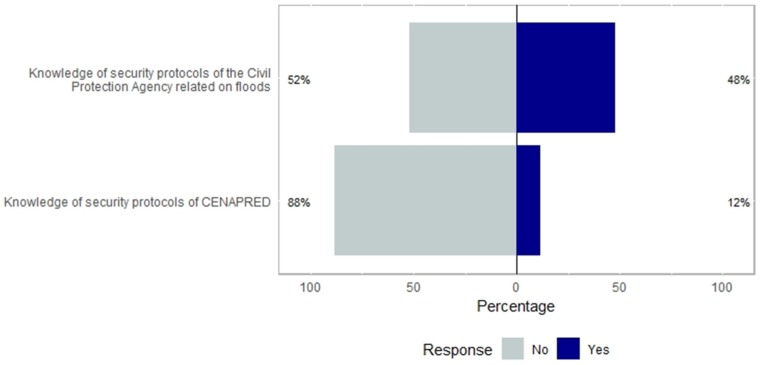
Centered bar plot generated with Likert with response distributions grouped by perceived knowledge of security protocols of the Civil Protection Agency related on floods and knowledge.

**Figure 4:**
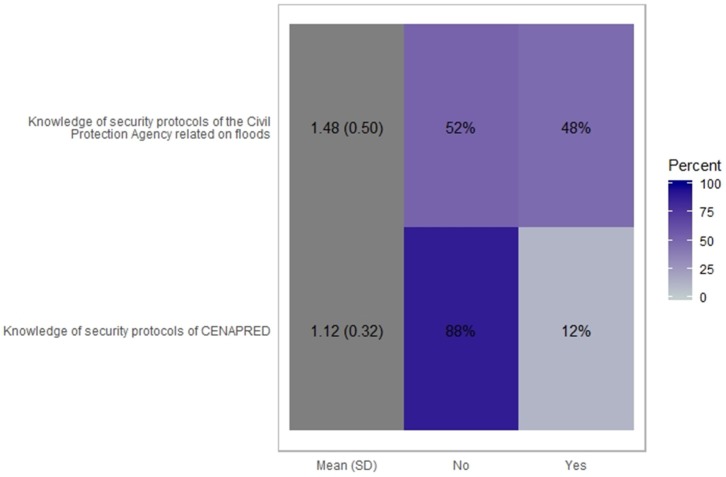
Graphic generated by percentage of perceived knowledge of security protocols of the Civil Protection Agency related on floods.

In our analysis presented in Figure 3 (in percentage), we show the responses to the two questions about the knowledge of the functions of the National Center for Disaster Prevention and the Civil Protection Agency protocols for hydrometeorological extreme events. The answer “yes or no” is observed and found in Figure 3 and Figure 4. We found that overall awareness was low, <40%, but with more respondents knowing about the flood policies of the civil protection agency than the protocols of CENAPRED (National Disaster Prevention Center in Mexico).

### 3.2. Perception of Availability of Drinking Water, Food, Urban Services, Drainage Services, Communication Services, Electricity and Public Transport Services During a Flash Flood Event

**Figure 5:**
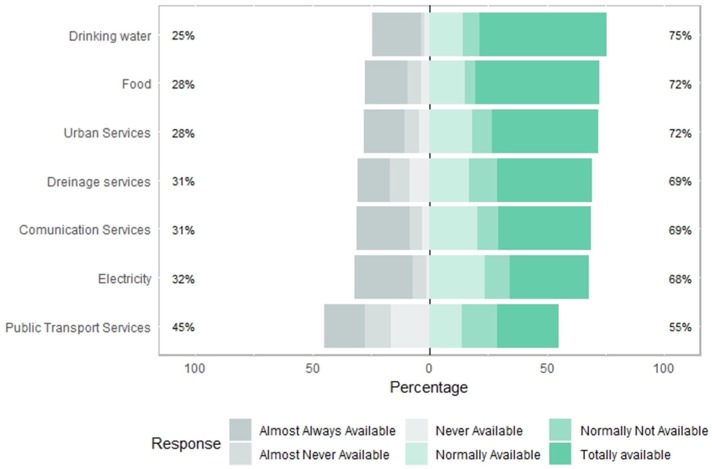
Centered bar plot generated with Likert with response distributions grouped by perceived availability of drinking water, food, urban services, drainage services, communication services, electricity and public transport services before a flood occurs and, if necessary, after it has occurred.

**Figure 6:**
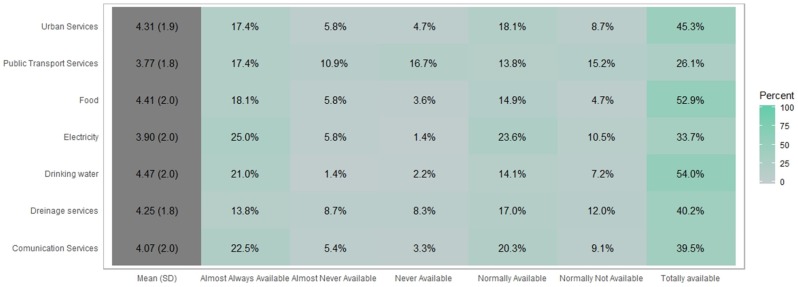
Graphic generated by percentage of perceived availability of drinking water, food, urban services, drainage services, communication services, electricity and public transport services before a flood occurs and, if necessary, after it has occurred.

Respondents were asked about the availability of drinking water, food, public (urban) services, drainage services, communication services, electricity, and public transport services to rate on a scale from 1 to 5. Figure 5 and Figure 6 show the results. 

### 3.3. Perception of Availability of Public Services during a Flash Flooding Event

**Figure 7:**
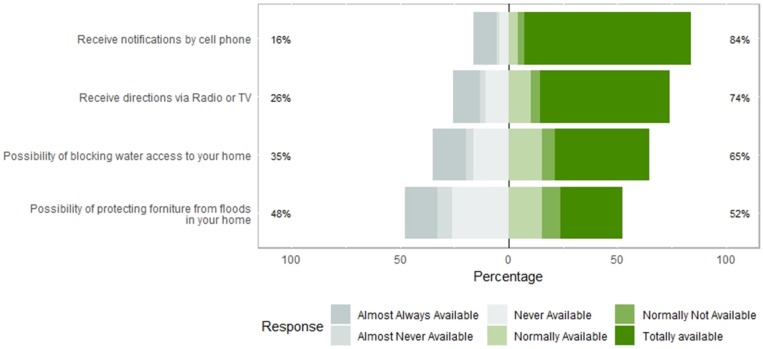
Centered bar plot generated with Likert with response distributions grouped by perceived possibility of blocking water access to homes, receive directions via radio/TV/cell phone and to protect furniture during a flash flooding event.

**Figure 8:**
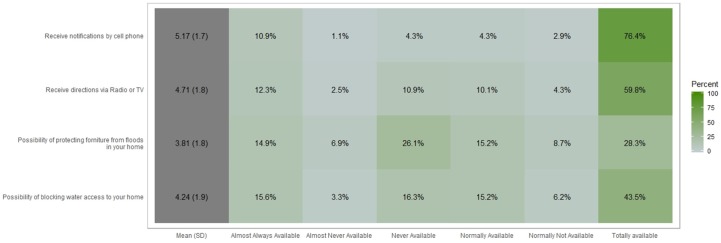
Graphic generated by percentage of perceived possibility of blocking water access to homes, receive directions via radio/TV/cell phone and to protect furniture during a flash flooding event.

Respondents were asked about availability to receive notifications by cell phone, radio or TV during a flash flood event. Also, the results from the possibility of blocking water access to homes and protecting it from floods are in Figure 7 and Figure 8 on a scale from 1 to 5. 

Table 1 presents statistics of our study sample, stratified by the three parts of the Questionnaire (Q1, Q2 and Q3).

**Figure 9:**
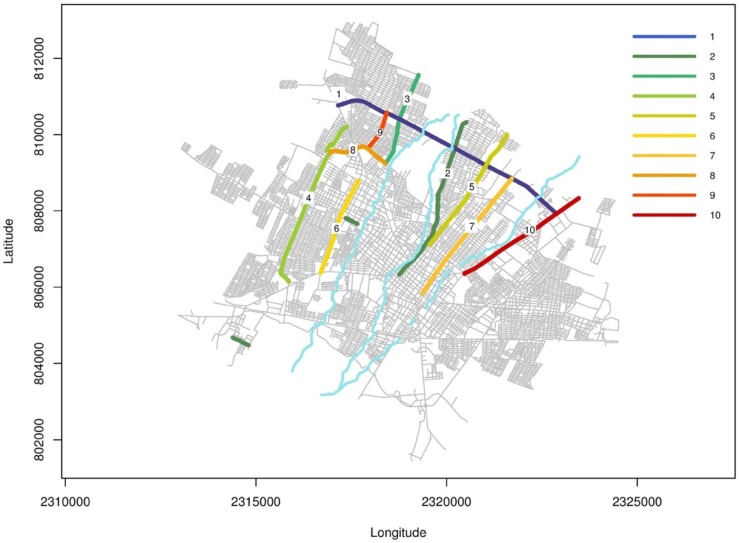
Map of the resilience index in the study area of Colima-Villa de Alvarez, Mexico. The column of lines in colors shows the resilience index per flooded zone [23].

In Figure 9, there is a map with the resilience indicated by the line from 1–10. On Table 2, there are the names of each street, affected the population, resilience index, identification color and the average level of resilience index. 

## 4. Discussion

Our findings suggest that most people susceptible to floods do not know the security protocols of the National Center for Disaster Prevention (88%) and only half (52%) knows the State Civil Protection Agency protocols for hydrometeorological extreme events. This is critical because these institutions in Mexico are responsible for making the right policies and react when a hazard occurs. This is a serious topic because all the surveyed population had experienced at least one flash flooding event. These findings show the urgent need of education for general people and more specifically for those more vulnerable. This has been raised by other studies [25,26,27], and raises the urgent need to make the vulnerable population aware of their risk and the policies regarding flooding in their area.

In Colima State there is an atlas risk map published that the State Government [28] in which there are hazards as flooding, pollution, earthquakes, and others. There is also another policy instrument on the risk that the Colima Municipality made [29] on risk with municipal scope. In these documents, sections related to flooding phenomena highlight the areas at risk under some specific scenarios, showing potential population affected and the impacts of the economic activities and the environmental variables of a series of rivers of the Colima-Villa de Alvarez zone. The hazards on flooding risk and vulnerability are shown in these maps, and tier for 2, 5, 10, 20 and 50 years is projected. So, the quantification of resilience (beyond risk) for that policy document is provided by our work.

Risk perception of flooding is a relevant issue [30,31,32,33]. Nowadays, to understand the situation of the vulnerable population knowledge on flooding risk is becoming more critical due to the lack of diverse and efficient communication platforms in developing countries and the different strategies to impact the most vulnerable. Even though public agencies or institutions exist, our findings suggest the need for more information and education of these populations. Many communications tasks can and should be undertaken before a disaster to improve preparedness. Some of these tasks represent common sense, while others may be more novel. Investing time and workforce now to improve an organization’s communications capacity can save time in disseminating key messages to minimize chaos and coordinate stakeholders once disaster strikes [34]. The most common cause of communications failures during disasters is the physical damage to devices or components that make up the network infrastructure [35]. Flooding can create a physical disturbance that have the power to do significant damage to cities and the vulnerable communications equipment that’s responsible for supporting these areas. Pre-preparedness and early warning systems are therefore critical.

The perception of the availability of public services during flash flooding events highlighted that 45.3% of the surveyed population perceive no loss of the availability of public services. After a flash flooding event, the Total Availability (TA) of public transport service availability is perceived 26.1%, this can be because of streets flooding, and debris, such as tree branches, trash, and stones blocking roads [36,37,38].

Lack of food during a flooding event is not a significant problem in the study area since more than a half vulnerable population (52.9%) perceived they could access food services. However, the other 47.1% have problems to access food, due to the closure of stores and markets. To have access to foods during a flooding phenomenon is critical [39,40]. This is a complex topic even in non-flood periods, because the Mexican population has low access to food [41] and even is entering the frontier of food security, in need for sustainable transversal policies linking food production and food consumption [42].

In terms of reducing the impacts of flooding, solutions such as blocking water access to homes were assessed. Almost Never Available (ANA), Never Available (NeverA), and Normally Not Available (NNA) (3.3%, 16.3%, and 6.2%) do not have control of the water access to their homes. This represents around 6,817 people approximately who can lose their belongings. The 41.7% (6.9%, 26.1%, and 8.7%) of the people who cannot protect the furniture in case of flooding, this represents 11,019 people that are susceptible to lose all furniture and belongings when a flood occurs. 76.4% of people can receive cellular notifications with total availability during and after a flooding event, and 59.8% can receive directions via radio and or TV. This point is important to another objective of the EWIN project [43] we are performing as other studies related to preventing floods alerting population [44,45,46]. 

Figure 9 shows the resilience index of the surveyed population in our study. Resilience on population to flooding has been documented [47,48] and this calculation is very useful in many ways [49,50,51], since the zones with minor resilience (zones 6 and 8) need to have a major effort from the Government to mitigate flooding risks and make programs for vulnerable people. Zones 1, 6, 7, 8 and 9 are below the scale of the average level of the resilience index by flood [24]; and therefore 12,253 people are most vulnerable to floods in the study area. Also, the results show that the population of Colima-Villa de Alvarez area 537,785 inhabitants [18] can be alerted and have a better response in preventing damages in a case of flooding. Because the results of the different levels of resilience is very specific to some streets that have had flash flooding events, is important to focus on what the data can teach us about flood risk, and how this can be applied to other areas of the world in order to prevent bigger flooding damages by flash flooding events.

The study of how individuals can prepare for flooding better as well as how private and public companies can mitigate the negative impacts from extreme weather events is important [52,53] due the fact that insurers optimize their catastrophe risk insurance policy design, portfolio, and risk transfer decisions within a context defined by homeowners, reinsurers, and government agencies using a credible assessment of natural disaster and the interactions among key stakeholders and between the two important risk management mechanisms of insurance and retrofit.

## 5. Conclusions

Global environmental change is placed in the context of system and community to help address the risks related to the environment. Numerous initiatives aim to incorporate resilience into urban planning practices. The purpose of this article is to open a conversation about urban resilience through the analysis of data obtained in the field. This study contributes to the production of different levels of perception and urban resilience in the Latin American perspective, and specifically in the study area of Colima-Villa de Alvarez, where the findings will help decision-makers to prioritize the immediate actions to protect the population from flash flooding events.

## Figures and Tables

**Table 1:** Statistics of the perception and resilience index to flooding in the study area of Colima-Villa de Alvarez, Mexico.

**Q1**	**Vars**	***n***	**Mean**	**sd**	**Med**	**Trimmed**	**Mad**	**Min**	**Max**	**Range**	**Skew**	**Kurtosis**	**se**
Knowledge of security protocols of CENAPRED	1	276	1.116	0.321	1	1.023	0	1	2	1	2.386	3.707	0.019
Knowledge of security protocols of the Civil Protection Agency related on floods	2	276	1.478	0.500	1	1.473	0	1	2	1	0.087	−2.000	0.030
**Q2**	**Vars**	***n***	**Mean**	**sd**	**Median**	**Trimmed**	**Mad**	**Min**	**Max**	**Range**	**Skew**	**Kurtosis**	**se**
Possibility of protecting furniture from floods in your home	1	276	3.873	1.775	4	3.964	2.224	1	6	5	−0.217	−1.273	0.107
Receive directions via Radio or TV	2	276	4.768	1.798	6	5.077	0	1	6	5	−1.140	−0.247	0.108
Possibility of blocking water access to your home	3	276	4.326	1.869	5	4.527	1.483	1	6	5	−0.680	−1.036	0.113
Receive notifications by cell phone	4	276	5.181	1.668	6	5.590	0	1	6	5	−1.805	1.616	0.100
**Q3**	**Vars**	***n***	**Mean**	**sd**	**Median**	**Trimmed**	**Mad**	**Min**	**Max**	**Range**	**Skew**	**Kurtosis**	**se**
Electricity	1	276	4.029	2.023	5	4.158	1.483	1	6	5	−0.562	−1.369	0.122
Drinking wáter	2	276	4.540	1.999	6	4.793	0	1	6	5	−0.996	−0.732	0.120
Communication Services	3	276	4.178	2.009	5	4.342	1.483	1	6	5	−0.656	−1.243	0.121
Public Transport Services	4	276	3.754	1.816	4	3.815	2.965	1	6	5	−0.177	−1.341	0.109
Drainage services	5	276	4.304	1.835	5	4.500	1.483	1	6	5	−0.680	−1.020	0.110
Food	6	276	4.511	1.979	6	4.757	0	1	6	5	−0.919	−0.872	0.119
Urban Services	7	276	4.402	1.921	5	4.622	1.483	1	6	5	−0.833	−0.913	0.116

**Table 2:** Resilience index and affected population on the study area of Colima-Villa de Alvarez, Mexico.

	Name of the Street	Affected Population	Resilience Index	ID Color	Resilience Index Medium
1	Tercer Anillo	1691	3.92	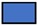	3.70 Scale of the average level of the resilience index by flood [23]
2	Venustiano Carranza	2945	3.53	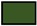	
3	Hidalgo	3494	3.74	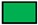	
4	Pablo Silva	4811	4.87	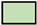	
5	Constitucion	1998	3.70	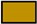	
6	Benito Juarez	2647	3.14	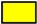	
7	Ignacio Sandoval	3754	3.54	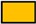	
8	Maria Ahumada	1362	3.18	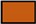	
9	Ayuntamiento	1545	3.68	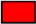	
10	Camino Real	2179	3.72	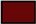	
	Total population	26,426			
